# 

**Published:** 1854-05

**Authors:** 


☞ Owing to the scarcity of paper and the breaking down of the mill that usually supplies us, this number of the Journal is delayed much beyond the usual time of publication.

### RECEIPTS

### FOR THE JOURNAL DURING THE MONTHS APRIL AND MAY

### Illinois.

| | |
|---|---|
| Dr. A. Hull, | $4 00 |
| " T. P. Mathews, | 5 00 |
| " G. P. Ransom | 2 00 |
| " N. Ray, | 2 00 |
| " J. H. Brown, | 2 00 |
| " H. C. Donaldson, | 2 00 |
| " W S Maus, | 6 00 |
| " Henry Bloxam, | 2 00 |
| " O. H. Martin, | 2 00 |
| " S. Shepard, | 2 00 |
| " H. H. Hall. | 2 00 |
| " John B. Glass, | 2 00 |
| " Charles Newton, | 2 00 |
| " A. S. Haskill, | 2 0 |
| " Fitch, | 6 00 |
| " D. Ward, | 2 00 |
| " C. D. Henton, | 2 00 |
| " H. H. Sexton, | 2 00 |
| " H. Hull, | 2 00 |
| " S C Hatch, | 2 00 |
| " J. M. Huggans, | 5 00 |
| " P B. McKay, | 4 00 |
| " Ehehardt, | 2 00 |
| " Algiers, | 4 00 |
| " Stephens, | 2 00 |
| " Cressy, | 6 00 |
| " J. B. Simpson, | 6 00 |
| " Robinson, | 2 00 |
| " McArthur, | 2 00 |
| " Smith. | 2 00 |
| " Snowdon | 2 00 |
| " Clark & Rice, | 2 00 |
| " R. McGee, | 4 00 |
| " G. T. Allen | 3 00 |
| " R McArthur, | 2 0 |
| " T. P. Montgomery, | 3 00 |
| " Miliken, | 2 00 |
| " R. Dunlop, jr., | 5 00 |
| " M Mason, | 4 00 |
| " Rawson & Hanse, | 4 00 |
| " L L Lake, | 2 00 |
| " J C Hinsey, | 5 00 |
| " Newton, | 4 00 |
| " S W Abbot | 6 50 |
| " T Leeds | 6 00 |

### Ohio.

| | |
|---|---|
| Dr. H P Wagner, | 6 00 |

### Indiana.

| | |
|---|---|
| Dr. A B Buchanan | 2 00 |
| " A Harrison, | 2 00 |
| " F B Cox, | 4 00 |
| " R A Perkins, | 2 00 |
| " M Bell | 2 00 |
| " D H Crouse, | 6 00 |
| " G A Durr, | 6 00 |
| " J S Baxter, | 2 00 |
| " H D Adams, | 2 00 |
| " J P Cobbs, | 2 00 |
| " E H Sulton | 1 00 |
| " W C Farrington, | 6 00 |
| " Mitchell & Reagan, | 2 00 |
| " A Davison | 2 00 |
| " L H Sovereign, | 4 00 |
| " L A Cass | 6 00 |
| " A J Minich | 2 00 |

### Wisconsin.

| | |
|---|---|
| Dr. W P McAllister. | 4 00 |
| " L B W Johnson, | 2 00 |
| " Wm Law, | 6 00 |
| " John R Goodnough, | 2 00 |
| " J H Turner, | 1 00 |
| " C Nettleson, | 2 00 |
| " Van Norstrand & Marks, | 2 00 |

### Iowa.

| | |
|---|---|
| Dr. Robert McGavern, | 2 00 |
| " John H Rice, | 2 00 |
| " A Boomer | 2 00 |
| " H W Hart, | 2 00 |

### Michigan.

| | |
|---|---|
| Dr. J F Williams, | $2 00 |
| " Tompkins & Garwood, | 2 00 |
| " Thomas Brayton, | 2 00 |
| " N D. Thomas. | 5 00 |
| " G B Davidson, | 1 00 |

### Louisiana.

| | |
|---|---|
| Dr. S W Purviance, | 2 00 |

### Pennsylnania.

| | |
|---|---|
| Dr, J Brelsford, | 6 00 |

### Minnesota Territory.

| | |
|---|---|
| Dr, J S Murphy, | 2 00 |

### California.

| | |
|---|---|
| Dr, J C Curtis & Club, | 5 00 |

